# 2,4,5-Triphenyl-1-(prop-2-en-1-yl)-1*H*-imidazole

**DOI:** 10.1107/S1600536813014104

**Published:** 2013-05-31

**Authors:** Mehmet Akkurt, Shaaban K. Mohamed, Adel A. E. Marzouk, V. M. Abbasov, Francisco Santoyo-Gonzalez

**Affiliations:** a Department of Physics, Faculty of Sciences, Erciyes University, 38039 Kayseri, Turkey; b Chemistry and Environmental Division, Manchester Metropolitan University, Manchester, M1 5GD, England; c Chemistry Department, Faculty of Science, Mini University, 61519 El-Minia, Egypt; d Pharmaceutical Chemistry Department, Faculty of Pharmacy, Al Azhar University, Egypt; e Mamedaliev Institute of Petrochemical Processes, National Academy of Sciences of Azerbaijan, Baku, Azerbaijan; f Department of Organic Chemistry, Faculty of Science, Institute of Biotechnology, Granada University, Granada, E-18071, Spain

## Abstract

In the title compound, C_24_H_20_N_2_, one of the ring C atoms and one of the ring N atoms are disordered over two sets of sites in a 0.615 (3):0.385 (3) ratio. The two parts of the disordered imidazole ring adopt an envelope conformation, with the undisordered ring N atom as the flap, displaced by −0.118 (6) and 0.226 (7) Å, respectively, in the two disorder components from the plane through the other ring atoms. The crystal structure features C—H⋯N hydrogen bonds and C—H⋯π inter­actions, which lead to the formation of infinite chains along [010].

## Related literature

For the biological significance of imidazole derivatives, see, for example: Kumar (2010 ▶); Castaño *et al.* (2008 ▶); Banfi *et al.* (2006 ▶); Bogle *et al.* (1994 ▶). For the synthesis and the structures of similar imidazoles, see: Mohamed *et al.* (2013*a* ▶,*b* ▶); Akkurt *et al.* (2013 ▶). For puckering parameters, see: Cremer & Pople (1975 ▶).
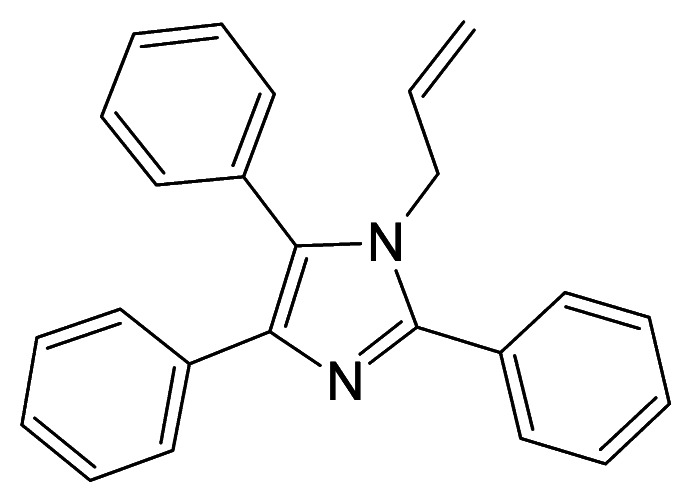


## Experimental

### 

#### Crystal data

C_24_H_20_N_2_
*M* *_r_* = 336.42
Monoclinic, 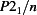

*a* = 10.362 (3) Å
*b* = 8.938 (2) Å
*c* = 19.387 (5) Å
β = 90.340 (5)°
*V* = 1795.5 (8) Å^3^
*Z* = 4
Mo *K*α radiation
μ = 0.07 mm^−1^
*T* = 100 K
0.14 × 0.14 × 0.003 mm

#### Data collection

Bruker SMART APEX CCD area-detector diffractometer
Absorption correction: refined from Δ*F* (*XABS2*; Parkin *et al.*, 1995 ▶) *T*_min_ = 0.990, *T*_max_ = 1.000
17127 measured reflections
3168 independent reflections
2442 reflections with *I* > 2σ(*I*)
*R*_int_ = 0.046

#### Refinement

*R*[*F* ^2^ > 2σ(*F* ^2^)] = 0.062
*wR*(*F* ^2^) = 0.183
*S* = 1.04
3168 reflections
299 parameters
12 restraints
H-atom parameters constrained
Δρ_max_ = 0.49 e Å^−3^
Δρ_min_ = −0.29 e Å^−3^

### 

Data collection: *SMART* (Bruker, 2001 ▶); cell refinement: *SAINT* (Bruker, 2001 ▶); data reduction: *SAINT*; program(s) used to solve structure: *SHELXS97* (Sheldrick, 2008 ▶); program(s) used to refine structure: *SHELXL97* (Sheldrick, 2008 ▶); molecular graphics: *ORTEP-3 for Windows* (Farrugia, 2012 ▶); software used to prepare material for publication: *WinGX* (Farrugia, 2012 ▶) and *PLATON* (Spek, 2009 ▶).

## Supplementary Material

Crystal structure: contains datablock(s) global, I. DOI: 10.1107/S1600536813014104/zp2005sup1.cif

Structure factors: contains datablock(s) shelxl. DOI: 10.1107/S1600536813014104/zp2005Isup2.hkl

Supplementary material file. DOI: 10.1107/S1600536813014104/zp2005Isup3.cml

Additional supplementary materials:  crystallographic information; 3D view; checkCIF report

## Figures and Tables

**Table 1:** Hydrogen-bond geometry (Å, °) *Cg*3 and *Cg*4 are the centroids of the C4–C9 and C10–C15 rings, respectively.

*D*—H⋯*A*	*D*—H	H⋯*A*	*D*⋯*A*	*D*—H⋯*A*
C17*A*—H17*A*⋯N1^i^	0.95	2.45	3.246 (8)	141
C21*A*—H21*A*⋯*Cg*3^ii^	0.95	2.98	3.888 (5)	160
C21*B*—H21*B*⋯*Cg*4^i^	0.95	2.99	3.914 (4)	163

Symmetry codes: (i) 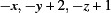; (ii) 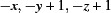.

**Fig. 1.**
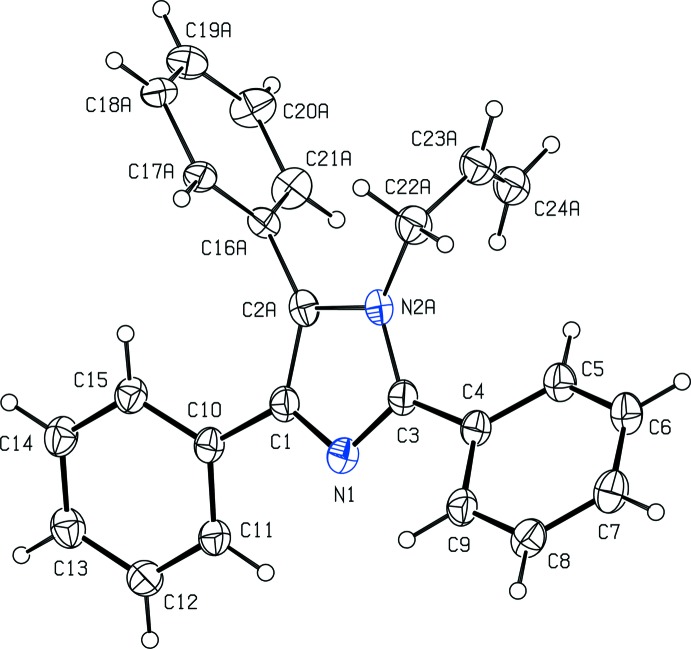
View of the major component of the disordered title compound with the atom numbering scheme. Displacement ellipsoids for non-H atoms are drawn at the 30% probability level.

**Fig. 2.**
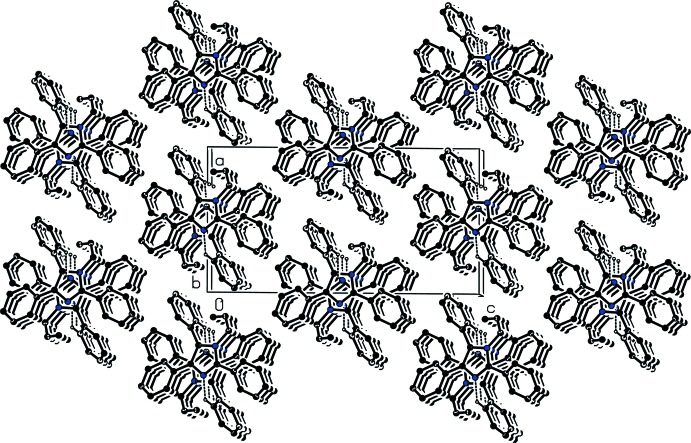
The packing diagram of the title compound viewing along the b axis. The hydrogen atoms not involved in hydrogen bonding and the minor component of the disorder have been omitted for clarity.

## supplementary crystallographic information

### Comment

The substituted imidazole derivatives are valuable in treatment of many systemic microbial infections and exhibit different types of pharmacological and biological activities (Kumar 2010). A number of substituted imidazoles, including clotrimazole, are selective inhibitors of nitric oxide synthase, which makes them interesting drug targets in inflammation, neurodegenerative diseases and tumors of the nervous system (Bogle *et al.*, 1994; Castaño *et al.*, 2008). Imidazoles also belong to the class of azole antifungals (Banfi *et al.*, 2006), which includes 1-vinyl imidazole, ketoconazole, miconazole, and clotrimazole. In this aspect we have prepared a series of tetrasubstituted imidazoles including the title compound as potential bio-active precursors. Herein, we report the single-crystal X-ray structure of 2,4,5-Triphenyl-1-(prop-2-en-1-yl)-1*H*-imidazole (I).

In the title compound (I), (Fig. 1), the two parts of the disordered imidazole ring adopt an envelope conformation [the puckering parameters (Cremer & Pople, 1975) are Q(2) = 0.076 (3) Å, φ(2) = 357 (4) ° for (N1/N2A/C1/C2A/C3), and Q(2) = 0.146 (4) Å, φ(2) = 180 (3) ° for (N1/N2B/C1/C2B/C3)]. The phenyl rings (C4–C9, C10–C15, C16A–C21A and C16B–C21B) makes dihedral angles of 35.91 (7), 18.14 (17), 85.0 (2) and 87.8 (2)°, respectively, with the mean plane of the imadazole ring (N1/N2A/C1/C2A/C3) and the corresponding angles are 18.8 (3), 35.3 (2), 85.7 (3) and 83.6 (3)°, respectively, for (N1/N2B/C1/C2B/C3). The bond lengths in (I) are within normal ranges and are comparable with those reported for the similar structures (Mohamed *et al.*, 2013*a*,*b*; Akkurt *et al.*, 2013).

In the crystal structure, molecules are linked by intermolecular C—H···N hydrogen bonds (Table 1, Fig. 2). In addition, C—H···π interactions contribute to the stabilization of the crystal packing.

### Experimental

The title compound was synthesized according to our reported method (Mohamed *et al.* 2013*a*) in 83% yield. Colourless plates suitable for X-ray analyses were obtained by slow evaporation of a solution of (I) in ethanol, m.p. 377–379 K.

### Refinement

All H atoms were placed in geometrically, with C—H = 0.95 and 0.99 Å, and refined as riding with *U*_iso_(H) = 1.2 *U*_eq_(C) of the parent atom. The carbon (C2) and nitrogen (N2) atoms which are adjacent at the imidazole ring and the phenyl (C16–C21) and propane (C22—C24) groups which attached to them respectively, are disordered over two sites (with the suffixes A and B) with an occupancy ratio of 0.615 (3):0.385 (3). The atoms of the disordered propane groups were set to equal each other by an EADP instruction. The disordered phenyl ring (C16B–C21B) was constrained to a rigid hexagon with the AFIX 66 instruction, and for the other atoms of disorder the SIMU and DELU instructions were used in the refinement procedure.

### Crystal data

**Table:**

C_24_H_20_N_2_	*F*(000) = 712
*M**_r_* = 336.42	*D*_x_ = 1.245 Mg m^−^^3^
Monoclinic, *P*2_1_/*n*	Mo *K*α radiation, λ = 0.71073 Å
Hall symbol: -P 2yn	Cell parameters from 2698 reflections
*a* = 10.362 (3) Å	θ = 2.2–21.5°
*b* = 8.938 (2) Å	µ = 0.07 mm^−^^1^
*c* = 19.387 (5) Å	*T* = 100 K
β = 90.340 (5)°	Plate, colourless
*V* = 1795.5 (8) Å^3^	0.14 × 0.14 × 0.003 mm
*Z* = 4	

### Data collection

**Table:**

Bruker SMART APEX CCD area-detector diffractometer	3168 independent reflections
Radiation source: sealed tube	2442 reflections with *I* > 2σ(*I*)
Graphite monochromator	*R*_int_ = 0.046
phi and ω scans	θ_max_ = 25.0°, θ_min_ = 2.1°
Absorption correction: part of the refinement model (Δ*F*) (*XABS2*; Parkin *et al.*, 1995)	*h* = −12→12
*T*_min_ = 0.990, *T*_max_ = 1.000	*k* = −10→10
17127 measured reflections	*l* = −23→23

### Refinement

**Table:**

Refinement on *F*^2^	Primary atom site location: structure-invariant direct methods
Least-squares matrix: full	Secondary atom site location: difference Fourier map
*R*[*F*^2^ > 2σ(*F*^2^)] = 0.062	Hydrogen site location: inferred from neighbouring sites
*wR*(*F*^2^) = 0.183	H-atom parameters constrained
*S* = 1.04	*w* = 1/[σ^2^(*F*_o_^2^) + (0.0987*P*)^2^ + 0.7593*P*] where *P* = (*F*_o_^2^ + 2*F*_c_^2^)/3
3168 reflections	(Δ/σ)_max_ < 0.001
299 parameters	Δρ_max_ = 0.49 e Å^−^^3^
12 restraints	Δρ_min_ = −0.29 e Å^−^^3^

### Special details

**Table:**

Geometry. Bond distances, angles *etc*. have been calculated using the rounded fractional coordinates. All su's are estimated from the variances of the (full) variance-covariance matrix. The cell e.s.d.'s are taken into account in the estimation of distances, angles and torsion angles
Refinement. Refinement on *F*^2^ for ALL reflections except those flagged by the user for potential systematic errors. Weighted *R*-factors *wR* and all goodnesses of fit *S* are based on *F*^2^, conventional *R*-factors *R* are based on *F*, with *F* set to zero for negative *F*^2^. The observed criterion of *F*^2^ > σ(*F*^2^) is used only for calculating -*R*-factor-obs *etc*. and is not relevant to the choice of reflections for refinement. *R*-factors based on *F*^2^ are statistically about twice as large as those based on *F*, and *R*-factors based on ALL data will be even larger.

### Fractional atomic coordinates and isotropic or equivalent isotropic displacement parameters (Å^2^)

**Table:**

	*x*	*y*	*z*	*U*_iso_*/*U*_eq_	Occ. (<1)
N1	−0.08400 (18)	0.74686 (19)	0.49897 (9)	0.0410 (6)	
N2A	0.1170 (5)	0.7384 (5)	0.5420 (2)	0.0353 (16)	0.615 (3)
C1	−0.0060 (2)	0.8077 (2)	0.45077 (12)	0.0403 (7)	
C2A	0.1231 (6)	0.8099 (6)	0.4794 (3)	0.0360 (17)	0.615 (3)
C3	−0.0081 (2)	0.6952 (2)	0.54940 (11)	0.0402 (7)	
C4	−0.0590 (2)	0.6243 (2)	0.61210 (11)	0.0394 (7)	
C5	0.0089 (2)	0.5150 (2)	0.64866 (11)	0.0437 (7)	
C6	−0.0425 (2)	0.4520 (3)	0.70782 (12)	0.0486 (8)	
C7	−0.1627 (3)	0.4953 (3)	0.73117 (12)	0.0515 (8)	
C8	−0.2316 (2)	0.6021 (3)	0.69493 (13)	0.0489 (8)	
C9	−0.1804 (2)	0.6654 (3)	0.63597 (13)	0.0454 (8)	
C10	−0.0566 (2)	0.8751 (2)	0.38715 (11)	0.0390 (7)	
C11	−0.1781 (2)	0.8326 (3)	0.36242 (12)	0.0441 (8)	
C12	−0.2281 (2)	0.8935 (3)	0.30231 (12)	0.0482 (8)	
C13	−0.1588 (2)	0.9992 (3)	0.26605 (12)	0.0496 (8)	
C14	−0.0392 (2)	1.0439 (3)	0.29035 (12)	0.0494 (8)	
C15	0.0118 (2)	0.9824 (2)	0.35016 (11)	0.0429 (7)	
C16A	0.2459 (4)	0.8604 (4)	0.44996 (18)	0.0334 (11)	0.615 (3)
C17A	0.2941 (6)	0.9947 (9)	0.4667 (4)	0.0396 (16)	0.615 (3)
C18A	0.4150 (3)	1.0459 (4)	0.43689 (18)	0.0403 (11)	0.615 (3)
C19A	0.4771 (4)	0.9553 (5)	0.39306 (19)	0.0470 (14)	0.615 (3)
C20A	0.4300 (4)	0.8172 (5)	0.3761 (2)	0.0543 (14)	0.615 (3)
C21A	0.3143 (5)	0.7684 (5)	0.4041 (2)	0.0500 (16)	0.615 (3)
C22A	0.2230 (4)	0.7339 (5)	0.5920 (2)	0.0505 (10)	0.615 (3)
C23A	0.3217 (4)	0.6142 (5)	0.5810 (2)	0.0505 (10)	0.615 (3)
C24A	0.3129 (6)	0.5098 (6)	0.5380 (2)	0.0505 (10)	0.615 (3)
C24B	0.3213 (17)	0.999 (2)	0.4606 (8)	0.060 (2)	0.385 (3)
C17B	0.2961 (4)	0.5016 (5)	0.5314 (2)	0.085 (4)	0.385 (3)
C18B	0.4132 (4)	0.4561 (4)	0.5598 (2)	0.0480 (19)	0.385 (3)
C19B	0.4774 (3)	0.5477 (5)	0.6067 (2)	0.058 (2)	0.385 (3)
C20B	0.4245 (4)	0.6848 (5)	0.6252 (2)	0.063 (3)	0.385 (3)
N2B	0.1125 (8)	0.7634 (8)	0.4574 (4)	0.039 (2)	0.385 (3)
C2B	0.1184 (10)	0.6892 (9)	0.5210 (5)	0.038 (3)	0.385 (3)
C16B	0.2432 (3)	0.6387 (5)	0.5499 (2)	0.0393 (19)	0.385 (3)
C23B	0.3136 (8)	0.8876 (10)	0.4214 (4)	0.060 (2)	0.385 (3)
C21B	0.3074 (4)	0.7303 (4)	0.5968 (2)	0.059 (3)	0.385 (3)
C22B	0.2197 (8)	0.7681 (9)	0.4076 (4)	0.060 (2)	0.385 (3)
H6	0.00520	0.37850	0.73250	0.0580*	
H7	−0.19750	0.45210	0.77180	0.0620*	
H8	−0.31430	0.63210	0.71050	0.0590*	
H9	−0.22900	0.73820	0.61130	0.0540*	
H5	0.09120	0.48350	0.63290	0.0530*	
H22B	0.26730	0.83200	0.59130	0.0610*	0.615 (3)
H23A	0.39730	0.61750	0.60890	0.0610*	0.615 (3)
H24A	0.23890	0.50240	0.50900	0.0610*	0.615 (3)
H24B	0.38020	0.43820	0.53430	0.0610*	0.615 (3)
H11	−0.22710	0.76080	0.38720	0.0530*	
H12	−0.31050	0.86250	0.28590	0.0580*	
H13	−0.19300	1.04080	0.22470	0.0600*	
H14	0.00840	1.11730	0.26590	0.0590*	
H15	0.09440	1.01370	0.36610	0.0510*	
H17A	0.24920	1.05690	0.49830	0.0480*	0.615 (3)
H18A	0.44920	1.14150	0.44820	0.0490*	0.615 (3)
H19A	0.55590	0.98800	0.37330	0.0560*	0.615 (3)
H20A	0.47620	0.75470	0.34530	0.0650*	0.615 (3)
H21A	0.28130	0.67260	0.39230	0.0600*	0.615 (3)
H22A	0.18590	0.72080	0.63850	0.0610*	0.615 (3)
H17B	0.25230	0.43900	0.49930	0.1020*	0.385 (3)
H18B	0.44940	0.36250	0.54720	0.0570*	0.385 (3)
H19B	0.55740	0.51670	0.62610	0.0700*	0.385 (3)
H20B	0.46830	0.74740	0.65720	0.0760*	0.385 (3)
H21B	0.27130	0.82390	0.60940	0.0710*	0.385 (3)
H22C	0.26510	0.67060	0.40850	0.0730*	0.385 (3)
H22D	0.18350	0.78200	0.36070	0.0730*	0.385 (3)
H23B	0.38800	0.87800	0.39330	0.0730*	0.385 (3)
H24C	0.25300	1.02150	0.49140	0.0730*	0.385 (3)
H24D	0.39560	1.06170	0.45950	0.0730*	0.385 (3)

### Atomic displacement parameters (Å^2^)

**Table:**

	*U*^11^	*U*^22^	*U*^33^	*U*^12^	*U*^13^	*U*^23^
N1	0.0465 (10)	0.0351 (10)	0.0414 (11)	−0.0013 (8)	0.0027 (9)	0.0000 (8)
N2A	0.046 (2)	0.028 (3)	0.032 (3)	0.004 (2)	−0.0003 (18)	0.0005 (16)
C1	0.0441 (13)	0.0320 (12)	0.0449 (13)	0.0033 (9)	0.0037 (10)	0.0038 (9)
C2A	0.049 (3)	0.029 (3)	0.030 (3)	0.005 (2)	−0.003 (2)	−0.0054 (18)
C3	0.0446 (13)	0.0327 (12)	0.0432 (13)	0.0008 (9)	0.0022 (10)	0.0040 (9)
C4	0.0460 (13)	0.0309 (11)	0.0413 (12)	−0.0001 (9)	0.0022 (10)	−0.0008 (9)
C5	0.0513 (14)	0.0391 (12)	0.0409 (12)	0.0048 (10)	0.0069 (10)	0.0014 (10)
C6	0.0609 (16)	0.0417 (13)	0.0433 (13)	0.0056 (11)	0.0074 (11)	0.0063 (10)
C7	0.0666 (16)	0.0457 (14)	0.0422 (13)	−0.0032 (12)	0.0094 (12)	0.0021 (11)
C8	0.0518 (14)	0.0391 (13)	0.0559 (15)	−0.0005 (11)	0.0143 (12)	−0.0039 (11)
C9	0.0502 (14)	0.0319 (12)	0.0540 (14)	0.0001 (10)	0.0025 (11)	0.0034 (10)
C10	0.0455 (13)	0.0305 (11)	0.0411 (12)	0.0009 (9)	0.0034 (10)	−0.0004 (9)
C11	0.0456 (13)	0.0334 (12)	0.0533 (14)	0.0013 (10)	0.0012 (11)	−0.0001 (10)
C12	0.0486 (14)	0.0419 (13)	0.0539 (14)	−0.0003 (11)	−0.0080 (11)	−0.0054 (11)
C13	0.0610 (15)	0.0465 (14)	0.0413 (13)	0.0024 (12)	−0.0080 (11)	0.0011 (11)
C14	0.0612 (16)	0.0450 (14)	0.0418 (13)	−0.0069 (12)	−0.0030 (11)	0.0060 (11)
C15	0.0500 (13)	0.0384 (12)	0.0401 (12)	−0.0043 (10)	−0.0033 (10)	0.0009 (10)
C16A	0.0321 (19)	0.041 (2)	0.0270 (18)	0.0065 (16)	0.0013 (15)	−0.0007 (15)
C17A	0.030 (3)	0.038 (2)	0.051 (3)	0.005 (2)	0.010 (2)	0.000 (2)
C18A	0.0308 (19)	0.050 (2)	0.040 (2)	−0.0044 (17)	0.0001 (15)	−0.0003 (17)
C19A	0.041 (2)	0.059 (3)	0.041 (2)	0.0007 (18)	−0.0045 (16)	−0.0068 (18)
C20A	0.050 (2)	0.063 (3)	0.050 (2)	0.000 (2)	0.0207 (19)	−0.020 (2)
C21A	0.064 (3)	0.048 (3)	0.038 (2)	−0.006 (2)	0.003 (2)	−0.0151 (18)
C22A	0.0504 (16)	0.061 (2)	0.0401 (14)	0.0030 (14)	0.0057 (13)	0.0122 (12)
C23A	0.0504 (16)	0.061 (2)	0.0401 (14)	0.0030 (14)	0.0057 (13)	0.0122 (12)
C24A	0.0504 (16)	0.061 (2)	0.0401 (14)	0.0030 (14)	0.0057 (13)	0.0122 (12)
C24B	0.060 (4)	0.073 (4)	0.048 (3)	−0.007 (3)	−0.011 (3)	0.010 (2)
C17B	0.080 (7)	0.040 (5)	0.136 (10)	−0.013 (5)	0.001 (7)	−0.001 (5)
C18B	0.033 (3)	0.066 (4)	0.045 (3)	−0.002 (3)	−0.002 (3)	0.004 (3)
C19B	0.060 (4)	0.055 (4)	0.060 (4)	−0.009 (3)	0.029 (3)	−0.015 (3)
C20B	0.058 (4)	0.069 (5)	0.062 (4)	−0.004 (4)	−0.031 (4)	−0.018 (4)
N2B	0.053 (4)	0.036 (4)	0.029 (4)	−0.008 (3)	0.001 (3)	0.003 (3)
C2B	0.052 (4)	0.024 (5)	0.039 (5)	−0.005 (4)	−0.004 (4)	0.000 (3)
C16B	0.037 (3)	0.050 (4)	0.031 (3)	−0.014 (3)	0.003 (3)	−0.008 (3)
C23B	0.060 (4)	0.073 (4)	0.048 (3)	−0.007 (3)	−0.011 (3)	0.010 (2)
C21B	0.085 (6)	0.052 (4)	0.040 (4)	0.007 (4)	0.004 (4)	−0.018 (3)
C22B	0.060 (4)	0.073 (4)	0.048 (3)	−0.007 (3)	−0.011 (3)	0.010 (2)

### Geometric parameters (Å, º)

**Table:**

N1—C1	1.353 (3)	C20A—C21A	1.389 (6)
N1—C3	1.334 (3)	C20B—C21B	1.390 (6)
N2A—C2A	1.373 (7)	C22A—C23A	1.496 (6)
N2A—C3	1.361 (6)	C22B—C23B	1.468 (12)
N2A—C22A	1.461 (6)	C23A—C24A	1.254 (6)
N2B—C2B	1.401 (12)	C23B—C24B	1.255 (19)
N2B—C22B	1.477 (11)	C5—H5	0.9500
N2B—C1	1.296 (8)	C6—H6	0.9500
C1—C2A	1.445 (7)	C7—H7	0.9500
C1—C10	1.467 (3)	C8—H8	0.9500
C2A—C16A	1.469 (7)	C9—H9	0.9500
C2B—C3	1.426 (10)	C11—H11	0.9500
C2B—C16B	1.477 (11)	C12—H12	0.9500
C3—C4	1.471 (3)	C13—H13	0.9500
C4—C5	1.395 (3)	C14—H14	0.9500
C4—C9	1.392 (3)	C15—H15	0.9500
C5—C6	1.387 (3)	C17A—H17A	0.9500
C6—C7	1.383 (4)	C17B—H17B	0.9500
C7—C8	1.382 (4)	C18A—H18A	0.9500
C8—C9	1.384 (4)	C18B—H18B	0.9500
C10—C11	1.397 (3)	C19A—H19A	0.9500
C10—C15	1.394 (3)	C19B—H19B	0.9500
C11—C12	1.384 (3)	C20A—H20A	0.9500
C12—C13	1.381 (3)	C20B—H20B	0.9500
C13—C14	1.382 (3)	C21A—H21A	0.9500
C14—C15	1.385 (3)	C21B—H21B	0.9500
C16A—C17A	1.339 (9)	C22A—H22A	0.9900
C16A—C21A	1.406 (6)	C22A—H22B	0.9900
C16B—C17B	1.390 (6)	C22B—H22C	0.9900
C16B—C21B	1.390 (6)	C22B—H22D	0.9900
C17A—C18A	1.457 (7)	C23A—H23A	0.9500
C17B—C18B	1.390 (6)	C23B—H23B	0.9500
C18A—C19A	1.341 (5)	C24A—H24A	0.9500
C18B—C19B	1.390 (6)	C24A—H24B	0.9500
C19A—C20A	1.367 (6)	C24B—H24C	0.9500
C19B—C20B	1.390 (6)	C24B—H24D	0.9500
			
C1—N1—C3	107.06 (18)	C6—C5—H5	120.00
C2A—N2A—C3	105.9 (4)	C5—C6—H6	120.00
C2A—N2A—C22A	124.1 (5)	C7—C6—H6	120.00
C3—N2A—C22A	129.4 (3)	C6—C7—H7	120.00
C2B—N2B—C22B	124.0 (8)	C8—C7—H7	120.00
C1—N2B—C2B	105.6 (7)	C7—C8—H8	120.00
C1—N2B—C22B	130.0 (7)	C9—C8—H8	120.00
N1—C1—C2A	107.2 (3)	C4—C9—H9	119.00
N2B—C1—C10	122.9 (4)	C8—C9—H9	119.00
N1—C1—C10	122.26 (19)	C10—C11—H11	120.00
N1—C1—N2B	112.2 (4)	C12—C11—H11	120.00
C2A—C1—C10	130.0 (3)	C11—C12—H12	120.00
N2A—C2A—C1	106.6 (5)	C13—C12—H12	120.00
N2A—C2A—C16A	122.1 (5)	C12—C13—H13	120.00
C1—C2A—C16A	131.1 (4)	C14—C13—H13	120.00
N2B—C2B—C3	106.7 (7)	C13—C14—H14	120.00
C3—C2B—C16B	132.1 (7)	C15—C14—H14	120.00
N2B—C2B—C16B	120.8 (8)	C10—C15—H15	120.00
C2B—C3—C4	129.6 (4)	C14—C15—H15	120.00
N1—C3—C4	122.82 (19)	C16A—C17A—H17A	120.00
N1—C3—C2B	105.6 (4)	C18A—C17A—H17A	120.00
N1—C3—N2A	112.5 (2)	C16B—C17B—H17B	120.00
N2A—C3—C4	123.8 (2)	C18B—C17B—H17B	120.00
C5—C4—C9	118.0 (2)	C17A—C18A—H18A	121.00
C3—C4—C5	122.63 (19)	C19A—C18A—H18A	121.00
C3—C4—C9	119.34 (19)	C17B—C18B—H18B	120.00
C4—C5—C6	120.6 (2)	C19B—C18B—H18B	120.00
C5—C6—C7	120.5 (2)	C18A—C19A—H19A	119.00
C6—C7—C8	119.4 (2)	C20A—C19A—H19A	119.00
C7—C8—C9	120.2 (2)	C18B—C19B—H19B	120.00
C4—C9—C8	121.2 (2)	C20B—C19B—H19B	120.00
C11—C10—C15	118.1 (2)	C19A—C20A—H20A	120.00
C1—C10—C11	119.59 (19)	C21A—C20A—H20A	120.00
C1—C10—C15	122.34 (19)	C19B—C20B—H20B	120.00
C10—C11—C12	120.9 (2)	C21B—C20B—H20B	120.00
C11—C12—C13	120.3 (2)	C16A—C21A—H21A	120.00
C12—C13—C14	119.5 (2)	C20A—C21A—H21A	120.00
C13—C14—C15	120.5 (2)	C20B—C21B—H21B	120.00
C10—C15—C14	120.7 (2)	C16B—C21B—H21B	120.00
C17A—C16A—C21A	119.3 (5)	N2A—C22A—H22B	108.00
C2A—C16A—C21A	120.5 (4)	N2A—C22A—H22A	108.00
C2A—C16A—C17A	120.3 (4)	H22A—C22A—H22B	107.00
C2B—C16B—C17B	121.1 (5)	C23A—C22A—H22A	108.00
C17B—C16B—C21B	120.0 (3)	C23A—C22A—H22B	108.00
C2B—C16B—C21B	118.9 (5)	N2B—C22B—H22D	109.00
C16A—C17A—C18A	120.4 (6)	H22C—C22B—H22D	108.00
C16B—C17B—C18B	120.0 (4)	C23B—C22B—H22C	109.00
C17A—C18A—C19A	118.6 (4)	C23B—C22B—H22D	109.00
C17B—C18B—C19B	120.0 (4)	N2B—C22B—H22C	109.00
C18A—C19A—C20A	121.7 (4)	C22A—C23A—H23A	117.00
C18B—C19B—C20B	120.0 (3)	C24A—C23A—H23A	117.00
C19A—C20A—C21A	119.8 (4)	C22B—C23B—H23B	112.00
C19B—C20B—C21B	120.0 (4)	C24B—C23B—H23B	112.00
C16A—C21A—C20A	120.2 (4)	H24A—C24A—H24B	120.00
C16B—C21B—C20B	120.0 (4)	C23A—C24A—H24A	120.00
N2A—C22A—C23A	115.9 (4)	C23A—C24A—H24B	120.00
N2B—C22B—C23B	113.7 (7)	C23B—C24B—H24C	120.00
C22A—C23A—C24A	125.4 (4)	C23B—C24B—H24D	120.00
C22B—C23B—C24B	136.7 (11)	H24C—C24B—H24D	120.00
C4—C5—H5	120.00		
			
C3—N1—C1—C2A	−8.2 (3)	N1—C3—C4—C5	150.5 (2)
C3—N1—C1—C10	179.56 (17)	N2A—C3—C4—C9	139.5 (3)
C1—N1—C3—N2A	9.1 (3)	C9—C4—C5—C6	−1.4 (3)
C1—N1—C3—C4	178.39 (17)	C3—C4—C5—C6	179.5 (2)
C3—N2A—C22A—C23A	105.6 (5)	C5—C4—C9—C8	1.2 (3)
C2A—N2A—C3—N1	−6.0 (4)	C3—C4—C9—C8	−179.6 (2)
C22A—N2A—C3—N1	165.4 (4)	C4—C5—C6—C7	0.7 (3)
C2A—N2A—C3—C4	−175.2 (3)	C5—C6—C7—C8	0.1 (4)
C22A—N2A—C3—C4	−3.8 (6)	C6—C7—C8—C9	−0.2 (4)
C3—N2A—C2A—C16A	−174.0 (4)	C7—C8—C9—C4	−0.4 (4)
C3—N2A—C2A—C1	0.6 (5)	C1—C10—C11—C12	−179.6 (2)
C22A—N2A—C2A—C1	−171.4 (4)	C11—C10—C15—C14	−0.5 (3)
C2A—N2A—C22A—C23A	−84.4 (6)	C1—C10—C15—C14	−179.8 (2)
C22A—N2A—C2A—C16A	14.0 (8)	C15—C10—C11—C12	1.1 (3)
N1—C1—C10—C11	−23.1 (3)	C10—C11—C12—C13	−0.8 (4)
N1—C1—C2A—N2A	4.7 (4)	C11—C12—C13—C14	−0.1 (4)
C2A—C1—C10—C15	−14.0 (4)	C12—C13—C14—C15	0.7 (4)
C2A—C1—C10—C11	166.6 (3)	C13—C14—C15—C10	−0.4 (3)
N1—C1—C2A—C16A	178.7 (5)	C2A—C16A—C17A—C18A	−179.2 (5)
C10—C1—C2A—N2A	176.1 (3)	C17A—C16A—C21A—C20A	−0.6 (7)
C10—C1—C2A—C16A	−9.9 (7)	C21A—C16A—C17A—C18A	1.0 (8)
N1—C1—C10—C15	156.25 (19)	C2A—C16A—C21A—C20A	179.6 (4)
N2A—C2A—C16A—C17A	−86.4 (7)	C16A—C17A—C18A—C19A	−0.6 (8)
N2A—C2A—C16A—C21A	93.4 (6)	C17A—C18A—C19A—C20A	−0.2 (6)
C1—C2A—C16A—C21A	−79.8 (6)	C18A—C19A—C20A—C21A	0.6 (6)
C1—C2A—C16A—C17A	100.4 (7)	C19A—C20A—C21A—C16A	−0.2 (6)
N1—C3—C4—C9	−28.6 (3)	N2A—C22A—C23A—C24A	−8.4 (7)
N2A—C3—C4—C5	−41.4 (4)		

### Hydrogen-bond geometry (Å, º)

Cg3 and Cg4 are the centroids of the C4–C9 and C10–C15 rings, respectively.

**Table:**

*D*—H···*A*	*D*—H	H···*A*	*D*···*A*	*D*—H···*A*
C17*A*—H17*A*···N1^i^	0.95	2.45	3.246 (8)	141
C24*A*—H24*A*···N2*A*	0.95	2.54	2.882 (8)	101
C21*A*—H21*A*···*Cg*3^ii^	0.95	2.98	3.888 (5)	160
C21*B*—H21*B*···*Cg*4^i^	0.95	2.99	3.914 (4)	163

Symmetry codes: (i) −*x*, −*y*+2, −*z*+1; (ii) −*x*, −*y*+1, −*z*+1.
